# ANDIS: an atomic angle- and distance-dependent statistical potential for protein structure quality assessment

**DOI:** 10.1186/s12859-019-2898-y

**Published:** 2019-06-03

**Authors:** Zhongwang Yu, Yuangen Yao, Haiyou Deng, Ming Yi

**Affiliations:** 1 Department of Physics, College of Science, Huazhong Agricultural University, Wuhan, 430070 China; 2 Institute of Applied Physics, Huazhong Agricultural University, Wuhan, 430070 China

**Keywords:** Statistical potential, Pair-wise interaction, Protein decoy set, Distance cutoff, Protein structure prediction

## Abstract

**Background:**

The knowledge-based statistical potential has been widely used in protein structure modeling and model quality assessment. They are commonly evaluated based on their abilities of native recognition as well as decoy discrimination. However, these two aspects are found to be mutually exclusive in many statistical potentials.

**Results:**

We developed an atomic ANgle- and DIStance-dependent (ANDIS) statistical potential for protein structure quality assessment with distance cutoff being a tunable parameter. When distance cutoff is ≤9.0 Å, “effective atomic interaction” is employed to enhance the ability of native recognition. For a distance cutoff of ≥10 Å, the distance-dependent atom-pair potential with random-walk reference state is combined to strengthen the ability of decoy discrimination. Benchmark tests on 632 structural decoy sets from diverse sources demonstrate that ANDIS outperforms other state-of-the-art potentials in both native recognition and decoy discrimination.

**Conclusions:**

Distance cutoff is a crucial parameter for distance-dependent statistical potentials. A lower distance cutoff is better for native recognition, while a higher one is favorable for decoy discrimination. The ANDIS potential is freely available as a standalone application at http://qbp.hzau.edu.cn/ANDIS/.

**Electronic supplementary material:**

The online version of this article (10.1186/s12859-019-2898-y) contains supplementary material, which is available to authorized users.

## Background

The primary mission in protein structure prediction is to develop accurate energy functions for conformational search [1–5], model refinement [6–9], and model quality assessment [10–12]. However, because of the big size, the flexibility and the presence of solvent molecules, proteins are still extremely difficult to model with physics-based potential [13, 14]. especially when quantum mechanical calculation is involved [15]. The knowledge-based potential [16–19], which is extracted from the experimental structures deposited in Protein Data Bank, has been playing an increasingly important role in protein structure prediction since its emergence in 1990s [20–22]. Varieties of structural features were used to derive knowledge-based potentials, such as residue solvent accessibility [23, 24], residue or atom contact [25, 26], atom-pair distance distribution [27–29], side-chain orientation [16, 30, 31] and so on. The Boltzmann law and probability theory are commonly employed to convert the observed frequencies of specific structural features into statistical potentials [17, 20].

To evaluate a potential function, basically the following two aspects need to be considered: (a) can the potential recognize native or near-native structure from non-native structures? (b) can the energy scores given by the potential well reflect the structural qualities of different prediction models? Both aspects can be assessed by applying the potential to various protein structure decoy sets [32–35]. In fact, the majority of statistical potentials were derived by optimizing both performances in native recognition and decoy discrimination [30, 36–38]. However, native recognition emphasizes the differences of overall structure quality between native and decoy structures (e.g., by maximizing the all-atom energy difference between the native structure and other non-native structures). While decoy discrimination generally focuses on the backbone differences among decoy structures (e.g., by enhancing the correlation of potential score with GDT_TS, TM-score etc.). They are actually in different levels (atomic and residual levels, respectively), thus the coupling of them would require a trade-off in potential optimization. Our previous work clearly indicates that the potential’s abilities of native recognition and decoy discrimination cannot be optimized simultaneously with the same parameter sets [39]. For protein structure modeling, the ability of decoy discrimination is more crucial. Commonly the energy function targeted to the modeling method is used. But for researchers who want to choose a better structure for biological analysis, the overall structure quality with native structure as the gold standard should be emphasized.

In this work, we developed an atomic angle- and distance-dependent (ANDIS) statistical potential for protein structure quality assessment. A total of 167 residue-specific, heavy atom types are considered. As done in GOAP potential [37], we define a local coordinate system for every heavy atom in protein structure based on the positions of the atom and two of its bonded neighboring atoms. The pair-wise interaction between atoms with distance < 15.0 Å and residue separation ≥7 are considered. 5 angles (4 polar angles and 1 dihedral angle) are calculated according to the relative orientation of local coordinate systems between the two interacted atoms. Since the angles are strongly associated with side-chain packing and hydrogen-bonding, the ANDIS potential naturally integrates the atomic distance-dependent and orientation-dependent interactions. The distance cutoff is designed to be adjustable from 7 Å to 15.0 Å. A lower distance cutoff (< 9.5 Å) is recommended for native recognition, and the energy of each atom-pair with distance below 9.5 Å is weighted based on the degree of mutual exposure. On the contrary, a higher distance cutoff (≥10 Å) is recommended for decoy discrimination, and a distance-dependent atom-pair potential with random-walk reference state [30] is combined with the angle energies to enhance the ability of decoy discrimination.

We benchmarked ANDIS with a comprehensive list of publicly available statistical potentials (Dfire [36], RW [30], GOAP [37], DOOP [40], etc.), via 632 protein structural decoy sets collected from diverse sources. The results indicate that ANDIS significantly outperforms other reported statistical potentials in terms of native structure recognition. The effects of different protein datasets and distance cutoffs on ANDIS’s performance are also comprehensively investigated. A detailed discussion is given below.

## Methods

### Experimental protein structures for calculating the potentials

A non-redundant structural dataset of 3519 protein chains were used for potential derivation. It was culled by PISCES [41] from Protein Data Bank with pairwise sequence identity < 20%, resolution < 2.0 Å and R-factor < 0.25 (only the structures determined by X-ray crystallography were considered). The original list from PISCES contains about 7000 protein chains. We excluded the proteins with incomplete, missing or nonstandard residues and the proteins with length < 30 or > 1000 residues. The dataset is publicly available at http://qbp.hzau.edu.cn/ANDIS/.

### Definition of distance-dependent angles

Various aspects of structural features (e.g., solvent accessibility, electrostatic interaction, contact, distance, torsional angle) can be used to derive statistical potential, with distance-dependent pair-wise interaction being the most commonly adopted. In ANDIS potential the atom-pairs with residue separation (in protein sequence) ≥ 7 and distance < 15.0 Å are considered. There are a total of 167 residue-specific, heavy (non-hydrogen) atom types in the 20 common amino acids. The distance between atom pair is divided into 29 bins (first bin is 0–2.2 Å, bin wide is 0.4 Å from 2.2 Å to 7.0 Å and 0.5 Å from 7.0 Å to 15.0 Å). ANDIS is designed to capture the structural characteristics embedded in the relative orientation of interacting atoms as well as in the distance distribution of atom-pairs.

**Fig. 1:**
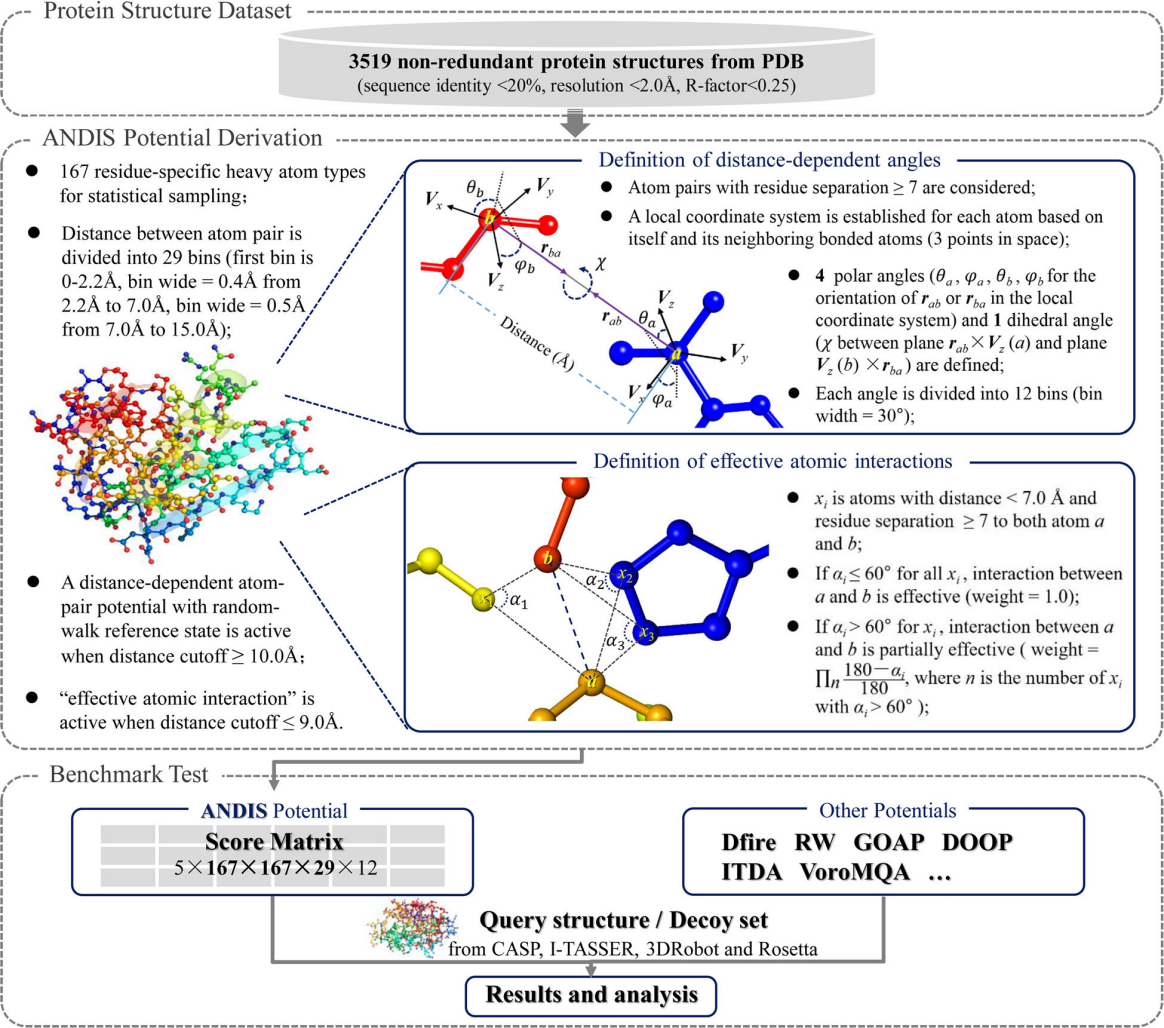
The flowchart of our studies. Step 1. PDB dataset preparation; Step 2. Potential derivation; Step 3. Benchmark test

As shown in Fig. 1, a local coordinate system is established for each atom based on itself and 2 neighboring bonded atoms (the next-neighbor, bonded atom is used if there is only one bonded heavy atom). To specify the relative orientation of the two coordinate systems, 5 distance-dependent angles are defined, including 4 polar angles (*θ*_*a*_, φ_*a*_, *θ*_*b*_, φ_*b*_ for the orientation of ***r***_*ab*_ or ***r***_*ba*_ in the local coordinate system) and 1 dihedral angle (*χ* between plane ***r***_*ab*_ × ***V***_*z*_ (*a*) and plane ***V***_*z*_ (*b*) × ***r***_*ba*_). A more detailed description of these angles is given by Zhou and Skolnick for their GOAP potential. [37]

The values of *θ*_*a*_, *θ*_*b*_, φ_*a*_, φ_*b*_ and *χ* are equally spitted into 12 bins. Thus the original size of the statistical matrix is 5 × 167 × 167 × 29 × 12. In statistics, we ignored the angle distributions (e.g., the second distance bin 2.2 Å–2.6 Å of atom-pair CYS N – PHE CE2 for angle φ_*a*_) whose occurrences were below 20 to ensure reasonable statistics.

### Definition of effective atomic interactions

In order to capture the pair-wise interactions that are more likely to be physically relevant, we consider only the “effective atomic interactions” in our potential [42]. As shown in Fig. 1, the physical exposure between atom *a* and *b* is evaluated by calculating the angle *α*_*i*_ (∠*ax*_*i*_*b*) for every atom *x*_*i*_ with distance < 7.0 Å to both atom *a* and *b*. A large angle *α*_*i*_ means that atom *a* and *b* are shielded by atom *x*_*i*_. Here we consider the interaction of atom *a* and *b* to be fully effective (assign weight = 1.0 in potential calculation) only when all angles *α*_*i*_ are equal to, or smaller than 60°. For the cases with *α*_*i*_ > 60°, we reduce the weight by weight = ∏_*i*_(180.0 − *α*_*i*_)/180.0 if residue separations between *x*_*i*_ and *a*, *b* are ≥2, and at least one of them are ≥7. This procedure can help eliminate the redundant and ineffective interactions in potential derivation and application.

### Calculation of ANDIS potential

The ANDIS potential is extracted from an experimental structural dataset of 3519 non-redundant protein chains based on the inverse Boltzmann equation [20]. We assume that the 5 angles (*θ*_*a*_, *θ*_*b*_, φ_*a*_, φ_*b*_ and *χ*) are independent of each other at the given distance so as to avoid insufficient statistics. Thus the angle potential can be written as:

1 $$ {\displaystyle \begin{array}{l}{E}^{AG}\left({\theta}_a,{\theta}_b,{\varphi}_a,{\varphi}_b,\chi \kern0.5em |{r}_{a,b}\right)=-{k}_{\mathrm{B}}T\ln \left[\frac{p^{OBS}\left({\theta}_a,{\theta}_b,{\varphi}_a,{\varphi}_b,\chi \kern0.5em |{r}_{a,b}\right)}{p^{REF}\left({\theta}_a,{\theta}_b,{\varphi}_a,{\varphi}_b,\chi \kern0.5em |{r}_{a,b}\right)}\right]\\ {}\kern15.5em \approx -{k}_{\mathrm{B}}T{\sum}_i\ln \left\{\frac{p^{OBS}\left[{angle}_i(s)\kern0.5em |{r}_{a,b}(d)\right]}{p^{REF}\left[{angle}_i(s)\kern0.5em |{r}_{a,b}(d)\right]}\right\}\end{array}} $$

where *k*_B_ and *T* are Boltzmann constant and Kelvin temperature, respectively. *r*_*a*, *b*_ is the distance between atom type *a* and *b*. *angle*_*i*_ is the angle *θ*_*a*_, *θ*_*b*_, φ_*a*_, φ_*b*_ or *χ*. *p*^*OBS*^[*angle*_*i*_(*s*) | *r*_*a*, *b*_(*d*)] and *p*^*REF*^[*angle*_*i*_(*s*) | *r*_*a*, *b*_(*d*)] are the observed and reference probabilities of *angle*_*i*_ falling into angle bin *s* at the given distance bin *d*. The initial count values for each angle bin are set to 0.1. Here we take the average observed value over 12 angle bins as the reference state, which means ${p}^{REF}\left[{angle}_i(s)\kern0.5em |{r}_{a,b}(d)\right]={\sum}_{s=1}^{12}{p}^{OBS}\left[{angle}_i(s)\kern0.5em |{r}_{a,b}(d)\right]/12$. The observed probabilities are calculated based on the entire structural dataset (3519 non-redundant X-ray structures). Eventually we can obtain an angle-based score matrix with the size of 5 × 167 × 167 × 29 × 12.

Since the best distance cutoff (*r*_*cut*_) is found to be highly depended on the evaluation criteria and the application environments, we make it an adjustable parameter from 7 Å to 15.0 Å for user. Generally, a lower distance cutoff is better for native recognition, while a higher one is favorable for decoy discrimination. The “effective atomic interaction” is employed to enhance the ability of native recognition when ${r}_{cut}\le 9.0\overset{\circ}{\mathrm{A}}$. For a distance cutoff of ≥10 Å, the distance-dependent atom-pair potential with random-walk reference state [30] (it yields an additional score matrix of 167 × 167 × 29) is combined with the angle potential to strengthen the ability of decoy discrimination. Therefore, the ANDIS energy score for a given protein sequence *S*_*q*_ with conformation *C*_*p*_ is calculated by

2 $$ E\left({S}_q,{C}_p\right)=\left\{\begin{array}{c}\sum \limits_{m=1}^{N\hbox{-} 1}\sum \limits_{n=m+1}^N{w}^{m,n}{E}^{AG}\left({\theta}_a^m,{\theta}_b^n,{\varphi}_a^m,{\varphi}_b^n,\chi \kern0.5em |{r}_{a,b}^{m,n}\right)\kern2.5em if\kern1.5em {r}_{cut}\le 9.5\overset{\circ}{\mathrm{A}}\kern1.5em \\ {}\sum \limits_{m=1}^{N\hbox{-} 1}\sum \limits_{n=m+1}^N\left(0.5\times {E}^{AG}\left({\theta}_a^m,{\theta}_b^n,{\varphi}_a^m,{\varphi}_b^n,\chi \kern0.5em |{r}_{a,b}^{m,n}\right)+{E}^{RW}\left({r}_{a,b}^{m,n}\right)\kern0.5em \right)\kern1.5em if\kern1em 10\overset{\circ }{\mathrm{A}}\le {r}_{cut}\le 15\overset{\circ }{\mathrm{A}}\kern0.5em \end{array}\right. $$

where *N* is the total number of heavy atoms in the protein chain *S*_*q*_. ${r}_{a,b}^{m,n}$ is the distance between atom pair *m* and *n* (corresponding to atom type *a* and *b*, respectively) observed in conformation *C*_*p*_. *r*_*cut*_ is the distance cutoff for ${r}_{a,b}^{m,n}$, which can be adjusted from 7.0 Å to 15.0 Å by user (Default value: 15.0 Å, and a lower value, e.g. 7.0 Å, is recommended if using ANDIS for native recognition). *w*^*m*, *n*^ is the weight for the energy score of atom pair *m* and *n* (${w}^{m,n}=1.0\kern0.5em if\kern0.5em {r}_{cut}=9.5\overset{\circ}{\mathrm{A}}\kern0.5em$), which is determined by the calculation of “effective atomic interactions” (see **Definition of effective atomic interactions**). ${E}^{RW}\left({r}_{a,b}^{m,n}\right)$ is the distance-dependent atom-pair potential with an ideal random-walk (RW) chain of a rigid step length as the reference state. We calculate RW potential based on the following equation:

3 $$ {E}^{RW}\left({r}_{a,b}\right)=-{k}_{\mathrm{B}}T\ln \frac{N^{OBS}\left({r}_{a,b}\right)}{\sum \limits_p^{N_{tot}}{\left(\frac{r_{a,b}}{r_{cut}}\right)}^2\frac{\sum_{n=1}^{L_p}\exp \left(-3{r}_{a,b}^2/2{nl}^2\right)/{n}^{3/2}}{\sum_{n=1}^{L_p}\exp \left(-3{r}_{cut}^2/2{nl}^2\right)/{n}^{3/2}}{N}_{a,b}^{OBS,p}\left({r}_{cut}\right)} $$

where *N*^*OBS*^(*r*_*a*, *b*_) is the total observed frequencies of atom type pairs (*a, b*) within a distance bin *r* to *r + Δr* in the experimental protein dataset. ${N}_{a,b}^{OBS,p}\left({r}_{cut}\right)$ is the observed frequencies of atom type pairs (*a, b*) within the distance bin of *r*_*cut*_ in protein *p*. *L*_*p*_ is the sequence length of protein *p*. *l* is Kohn length. *N*_*tot*_ is the total number of proteins in the experimental dataset. Only atom pairs with residue separation ≥7 are considered. More information about RW potential can be found in the original work by Zhang and Zhang [30].

### Decoy datasets for benchmark test

We collected hundreds of decoy sets (each set includes a native structure as well as a bunch of structural decoys) from diverse sources for benchmarking the ANDIS potential (see Table 1). The CASP5–8 decoy sets contain a total of 2759 structures for 143 proteins, which were collected from CASP5-CASP8 experiments by Rykunov and Fiser [43]. The CASP10–13 decoy sets were directly downloaded from http://predictioncenter.org/download_area/. We selected and trimmed these decoy sets based on the following procedure: (i) the prediction sets for targets without experimental structures are removed; (ii) the prediction sets whose target experimental structures are sequentially non-consecutive are removed; (iii) all non-first prediction models (the second to fifth models of predictors) are removed; (iv) the prediction models whose sequences are non-consecutive or shorter than the corresponding experimental structure are removed; (v) all prediction models are trimmed to keep them identical in sequence to the corresponding experimental structure. As a result, the final decoy sets include 175 target proteins (a total of 13,474 structures). The CASP10–13 decoy sets are publicly available at http://qbp.hzau.edu.cn/ANDIS/.

**Table 1:** Performance comparison in native recognition

Decoy sets	CASP5–8	CASP10–13	I-TASSER	3DRobot	Rosetta	No. total^d^
No. of targets^a^	143 (2759)	175 (13,474)	56 (24,707)	200 (60,200)	58 (5858)	632 (106,998)
Dfire^b^	64 (0.61)	56 (0.72)	43 (2.80)	1 (0.83)	22 (1.55)	186 (0.99)
RW	65 (1.01)	36 (0.86)	**53** (4.42)	0 (−0.30)	20 (1.48)	174 (0.90)
GOAP	106 (1.67)	89 (1.62)	45 (4.98)	94 (1.85)	45 (3.38)	379 (2.16)
DOOP	135 (1.96)	121 (1.99)	52 (6.18)	197 (3.53)	50 (3.91)	555 (3.02)
ITDA	71 (1.15)	117 (1.67)	52 (4.98)	196 (3.83)	**53** (3.52)	489 (2.70)
VoroMQA	132 (2.00)	111 (1.77)	48 (5.11)	114 (1.89)	43 (3.09)	448 (2.28)
SBROD	88 (1.62)	119 (2.32)	33 (3.25)	49 (1.76)	42 (3.02)	331 (2.13)
AngularQA	59 (1.26)	24 (1.11)	29 (1.82)	9 (0.99)	2 (0.12)	123 (1.08)
ANDIS^c^	**138** (**2.16**)	**129** (**2.32**)	47 (**6.45**)	**200** (**4.99**)	50 (**4.27**)	**564** (**3.67**)

^a^The total number of structures (including native structures) are given in parentheses

^b^The number of proteins whose native structure is given the lowest energy score by the potential are listed outside the parentheses. The average Z-scores of native structures are listed in parentheses. Z-score is defined as (<*E*_*decoy*_> − *E*_native_)/*δ*, where *E*_native_ is the energy score of native structure, <*E*_*decoy*_> and *δ* are respectively the average and the standard deviation of energy scores for all decoys in the set. But Z-score for VoroMQA energy score is calculated by (*E*_native_ −  < *E*_*decoy*_>)/*δ*, so that Z-scores of native structures for all potentials are “the higher the better”

^c^Calculation is based on a distance cutoff of 7.0 Å

^d^Z-scores are calculated by averaging over all 632 decoy sets

Moreover, we also used other three groups of decoy sets generated by some specific modeling methods. The I-TASSER decoy sets comprise of 56 non-redundant proteins (a total of 24,707 structures) whose structure decoys were generated by I-TASSER Monte Carlo simulations [44] and refined by GROMACS4.0 MD simulation [45]. The 3DRobot decoy sets were generated by a specialized decoy generating method we previously developed [35], which include 200 non-redundant proteins (a total of 60,200 structures). The Rosetta decoy sets include a total of 5858 structures for 58 proteins, which were generated by Rosetta ab initio structure prediction [46].

### Other potentials for benchmark comparison

We benchmarked ANDIS with other 8 state-of-the-art potentials. Two of them (Dfire [36] and RW [30]) are purely distance-dependent atom-pair statistical potentials with different analytical assumptions of reference state. GOAP [37] depends on the relative orientation of the planes associated with each heavy atom in interacting pairs, which combines Dfire with an angle-dependent potential. ITDA [47] integrates the distance-dependent atom-pair potential with a new component for estimating the backbone conformational entropies. VoroMQA [38] combines the idea of statistical potentials with the use of interatomic contact areas instead of distances. Contact areas, derived using Voronoi tessellation of protein structure, are capable of capturing both explicit interactions between protein atoms and implicit interactions of protein atoms with solvent. The other 3 potentials (DOOP [40], SBROD [48] and AngularQA [49]) employ machine learning methods to different extent. DOOP is a neural network-based potential with distance distributions of different atom pairs as input features. It also includes a torsion potential term which describes the local conformational preference. SBROD is trained based on Ridge Regression with four different structural features: residue-residue orientations, contacts between backbone atoms, hydrogen bonding, and solvent-solute interactions. AngularQA is derived based on Long Short-Term Memory (LSTM) network with the angles between residues being the core features. Like ANDIS, all the 8 potentials are single-model quality assessment methods.

## Results

### Effects of distance cutoff on ANDIS’s performance

**Fig. 2:**
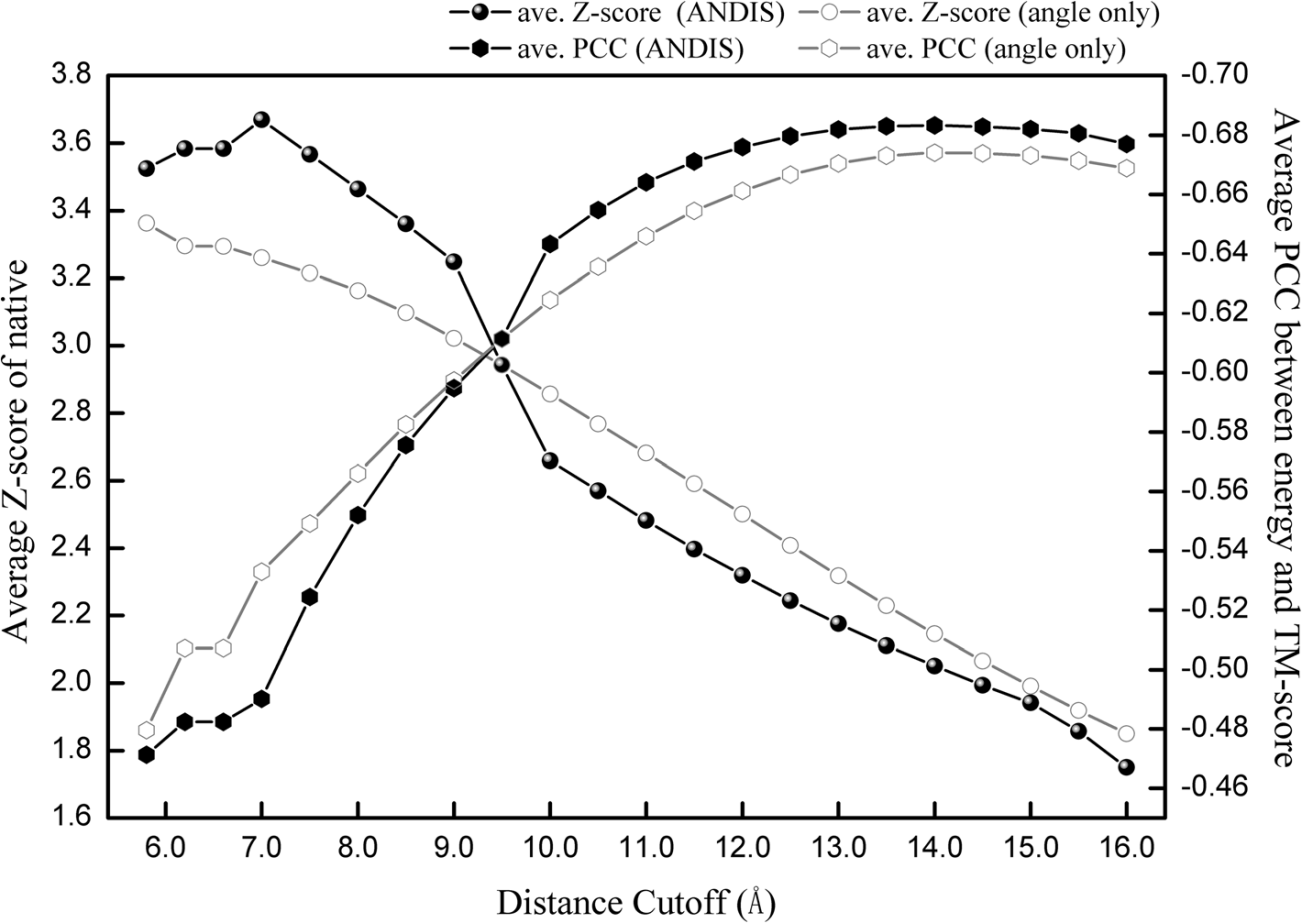
Effects of distance cutoff on ANDIS’s performance. The results are averaged over all 632 structural decoy sets. “angle only” refers to the pure angle potential without involvement of “effective atomic interaction” and distance-dependent atom-pair potential. Since lower energy score (higher TM-score) is desired, the value of PCC is negative, the lower the better

Distance cutoff is one of the most essential parameter for distance-dependent potentials. A series of distance cutoffs (from 5.8 Å to 16.0 Å) were tested to derive different versions of ANDIS potential. Figure 2 shows their average performance over all 632 decoy sets. Potential based on distance cutoff of around 7.0 Å achieves the highest average Z-score (of native structure). Afterwards, the average Z-score decreases linearly with the increase of distance cutoff. However, the average PCC (between ANDIS energy and TM-score) varies with distance cutoff in the opposite trend. These results indicate that the potential’s abilities of native recognition and decoy discrimination cannot be optimized simultaneously with the same distance cutoff. Generally, a lower distance cutoff is better for native recognition, while a higher one is favorable for decoy discrimination. But the optimal distance cutoff for decoy sets from different sources may vary. As shown in Additional file 1: Figure S1, the best cutoff of native recognition for I-TASSER decoy sets is 9.0 Å, and the best cutoff of decoy discrimination for 3DRobot decoy sets is 10.0 Å. Therefore, ANDIS provides distance cutoff as an adjustable parameter from 7.0 Å to 15.0 Å with bin-width of 0.5 Å. The default value is set to 15.0 Å in favor of decoy discrimination, and 7.0 Å is recommended for native recognition.

Since the “effective atomic interaction” is beneficial for native recognition but unhelpful for decoy discrimination, we include it only when a lower distance cutoff (≤ 9.0 Å) is adopted. As shown in Fig. 2, the average Z-score is significantly improved compared with that of angle potential only. The results for cases with higher distance cutoff (≥ 10.0 Å) also demonstrate a remarkable promotion in decoy discrimination achieved by incorporation of the distance-dependent atom-pair potential with random-walk reference state.

Moreover, we also checked the distance cutoffs used by the distance-dependent potentials listed in Table 1 (Dfire, RW, GOAP and DOOP), and found that most of them are around 15 Å, except that of DOOP (6.5 Å). This could provide a possible explanation for DOOP’s outstanding performance in native recognition.

### Performance comparison in native recognition

We applied ANDIS as well as other 8 potentials on the 632 decoy sets from CASP experiments [50], I-TASSER [30], 3DRobot [35] and Rosetta [46]. Table 1 summarizes the performances of different potentials in native recognition (recognize the native structure among a set of structural decoys). ANDIS (distance cutoff of 7.0 Å is used) recognizes 564 native structures (success rate is about 90%) and achieves an average Z-score of 3.67 over all decoy sets, which is remarkably better than that of the other eight potentials. For the CASP5–8 [43], CASP10–13 and 3DRobot decoy sets, ANDIS has the best performances. For I-TASSER and Rosetta decoy sets, ANDIS fails to achieve the best success rate, but still has the best Z-score.

The atomic distance-dependent pair-wise potentials, Dfire and RW, perform much worse than other potentials. Although their capabilities for native recognition can be remarkably improved by adjusting the distance cutoff and residue interval [39], they failed to outperform DOOP and ANDIS (data not shown). GOAP significantly outperforms Dfire and RW, but still has large gaps compared with other 4 potentials. The neural network-based potential DOOP (with distance cutoff of 6.5 Å) is the only one with comparable performance to ANDIS. Moreover, ITDA and VoroMQA, the two recently developed statistical potentials, both underperform DOOP in native recognition. However, ITDA achieves the best success rate (53 out of 58) on Rosetta decoy sets. The other two machine learning-based methods, SBROD and AngularQA, perform much worse than DOOP in native recognition, which is possibly because they are mainly designed for decoy ranking.

### Performance comparison in decoy discrimination

The more practical use of statistical potential is to discriminate between good and bad structural decoys. Table 2 summarizes the performances of different potentials in decoy discrimination. We evaluate the ability of decoy discrimination based on the average Pearson’s correlation coefficient (PCC) between energy score and TM-score, as well as the 20% enrichment which measures the relative occurrence of the most accurate (by TM-score) 20% decoys among the 20% best scoring (by potential) decoys. The outstanding performances of SBROD on CASP decoy sets help it achieves the best average performances over all decoy sets. However, its performances on the rest three groups of decoy sets are far worse than those of other methods (except AngularQA). In fact, SBROD are trained directly based on CASP5-CASP10 datasets, which probably brings it an inherent bias to CASP decoy sets. ANDIS achieves both the best average PCC (− 0.681) and the best average 20% enrichment (2.83) over all 632 decoy sets (except SBROD). The performances of VoroMQA are relatively close to that of ANDIS. GOAP outperforms all other potentials on 3DRobot decoy sets. In fact ANDIS is able to surpass GOAP on 3DRobot decoy sets if a distance cutoff between 10.0 Å to 13.0 Å is adopted (e.g., the average PCC and 20% enrichment on 3DRobot decoy sets are 0.910 and 4.14 when distance cutoff is set to 10.0 Å). DOOP and ITDA, which are outstanding in native recognition, perform noticeably worse than other potentials in decoy discrimination (except AngularQA). The bad performances of AngularQA are probably because it is mainly designed to serve as an energy component, not a standalone QA method.

**Table 2:** Performance comparison in decoy discrimination

Decoy sets	CASP5–8	CASP10–13	I-TASSER	3DRobot	Rosetta	Average^e^
Dfire^b^	−0.548 (2.16)	−0.441 (2.01)	− 0.480 (**1.62**)	−0.860 (3.77)	− 0.366 (1.97)	−0.594 (2.56)
RW	−0.550 (2.16)	−0.462 (2.01)	− 0.476 (1.59)	−0.863 (3.80)	− 0.361 (1.95)	−0.601 (2.57)
GOAP	−0.607 (2.66)	−0.550 (2.13)	− 0.473 (1.61)	**−0.900** (**4.04**)	**− 0.406** (1.99)	−0.654 (2.79)
DOOP	−0.442 (1.95)	−0.415 (1.90)	− 0.333 (1.41)	−0.874 (4.00)	− 0.285 (1.67)	−0.547 (2.51)
ITDA	−0.392 (2.03)	−0.452 (2.03)	− 0.431 (1.54)	−0.841 (3.70)	− 0.302 (1.68)	−0.545 (2.48)
VoroMQA^c^	0.665 (2.66)	0.628 (2.26)	0.450 (1.44)	0.893 (3.91)	0.366 (1.86)	0.680 (2.76)
SBROD^c^	**0.793** (**3.06**)	**0.831** (**2.26**)	0.397 (1.49)	0.857 (3.34)	0.270 (1.62)	**0.741** (2.66)
AngularQA^c^	0.441 (0.122)	0.426 (0.304)	0.323 (0.579)	0.543 (0.224)	0.042 (0.961)	0.422 (0.32)
ANDIS^d^	−0.663 (2.80)	−0.607 (2.18)	**−0.503** (1.59)	− 0.891 (3.95)	−0.401 (**2.05**)	− 0.681 (**2.83**)

^a^the native structures in the decoy sets are ignored when calculating PCC and “20% enrichment”

^b^The average Pearson’s correlation coefficient between energy and TM-score (PCC) is listed outside the parentheses. The average value of 20% enrichment is listed in parentheses. “20% enrichment” means the relative occurrence of the most accurate (by TM-score) 20% models among the 20% best scoring (by potential) models compared to that for the entire decoy set. The possible value of 20% enrichment ranges from 0 to 5, the higher the better

^c^Since the energy scores of VoroMQA, SBROD and AngularQA are the higher the better, the PCC between them and TM-score is positive

^d^Calculation is based on a distance cutoff of 15.0 Å

^e^by averaging over all 632 decoy sets

Calculation by GDT_TS (instead of TM-score) came up with very similar results (data not shown).

## Discussion

### Effects of protein dataset on ANDIS’s performance

**Fig. 3:**
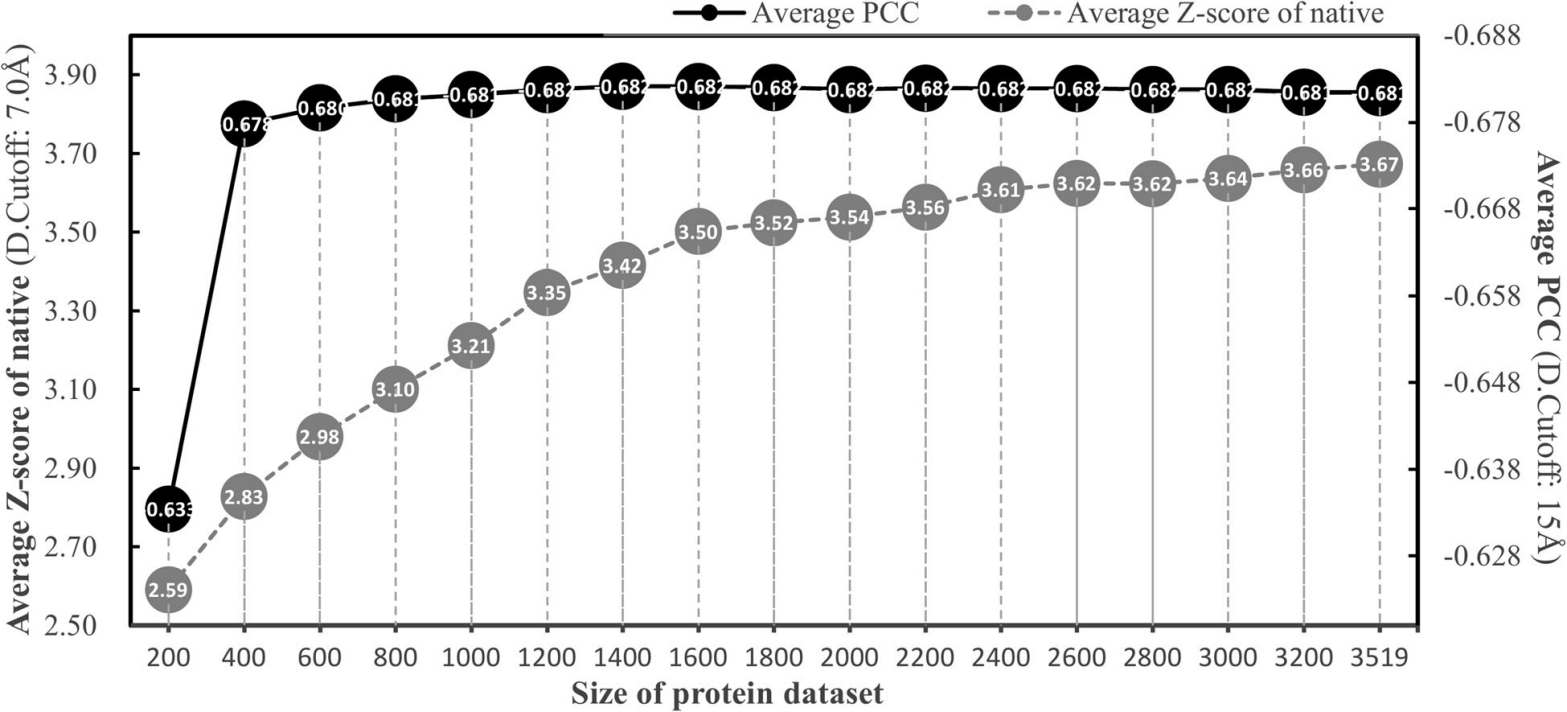
Overall effects of dataset size on ANDIS’s performance. ANDIS is re-extracted based on different number of structures from the original dataset (3519 structures)

By the beginning of 2018, the total number of structures deposited in the Protein Data Bank [51] has almost reached 140,000. The size and scope of protein dataset are no longer a problem for potential derivation. To demonstrate the correlation between dataset size and ANDIS’s performance, we derived ANDIS based on different number of protein structures from the dataset (3519 X-ray structures). As shown in Fig. 3, the average Z-score of native increases with the size of protein dataset, faster when the dataset is relatively small (e.g., < 1200), stabilized gradually when the dataset size exceeds 2000. However, the average PCC is very insensitive to the size of dataset. It is noteworthy that the potential based on only 400 structures can already achieve an average PCC very close to the optimal. This implies that the rest 3000 structures actually have very little contribution to promote potential’s ability of decoy discrimination. The same procedure was also conducted on other datasets listed in Additional file 1: Figure S2, similar trends were observed. In general, a dataset with around 3000 structures is adequate for ANDIS to obtain the optimal or near-optimal performance in native recognition.

Moreover, on what basis should a protein dataset be determined, and how does the choice of dataset affect potential’s performances? Here we prepared a series of structure datasets according to the pre-compiled PDB lists for various parameter sets (resolution, sequence identity, etc.) from PISCES [41]. We derived the ANDIS potential based on different datasets and summarized the test results in Additional file 1: Figure S2. It is easy to see that the performance variation brought by dataset with different parameter sets is very limited. There are almost no changes on average PCC for all 5 groups of decoy sets. The average Z-score for 3DRobot decoy sets increases slightly with the decrease of dataset size, but reverse trends can be seen for I-TASSER and Rosetta decoy sets. In fact, results based on datasets with size > 3000 are relatively stable.

### What kind of native structures are hard to be recognized?

**Fig. 4:**
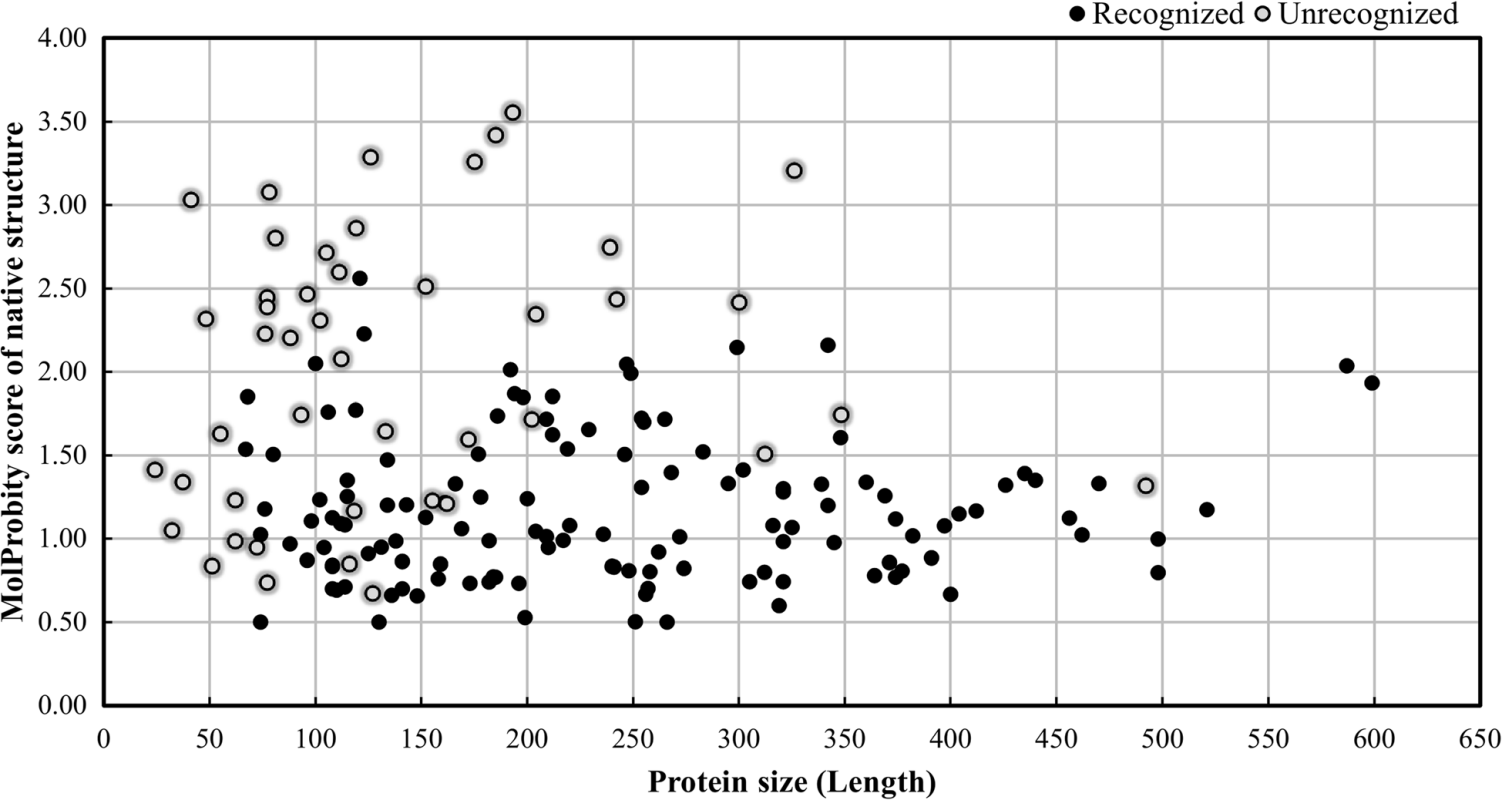
The protein size and MolProbity score for native structures in CASP10-13 decoy sets. ANDIS recognized 129 (out of 175) native structures in CASP10-13 decoy sets. The 46 unrecognized native structures are highlighted by shade open circles

Although 90% of native structures are successfully recognized by ANDIS, what are the other unrecognized 10%? We checked all the 58 unrecognized native structures, and found that their average length is significantly lower than that of the recognized. We also calculated the MolProbity score [52] of native structure. It is a well-known metric for estimating the physical reasonableness of protein structure. Figure 4 shows the length and MolProbity score of all 175 native structures in CASP10–13 decoy sets. We can see that all 9 native structures with length < 65 residues and 75% (24 out of 32) of native structures with MolProbity score > 2.0 are not recognized by ANDIS. Quite the contrary, more than 90% of native structures with length > 65 and MolProbity score < 2.0 are successfully recognized by ANDIS. Since higher MolProbity score implies worse structural quality (or lower resolution), these observations indicate that the hard targets for native recognition have a certain degree of commonality. In another sense, for the target protein of small size (or target protein whose experimental structure has relatively low resolution), current prediction methods are capable of generating protein models comparable to the experimental structure. Furthermore, all native structures in I-TASSER and Rosetta decoy sets are small proteins with average lengths of 80 residues and 83 residues, respectively. There is no evident difference in length between the recognized and the unrecognized native structures from them. But the average MolProbity scores of the unrecognized native structures from I-TASSER and Rosetta decoy sets are 2.386 and 2.506 respectively, much larger than those of the recognized native structures from them (1.223 and 1.771, respectively). Similar results are observed in CASP5–8 decoy sets. In fact all the 5 unrecognized native structures from CASP5–8 decoy sets are ranked second by ANDIS, only inferior to one prediction model.

## Conclusions

Our study demonstrates that distance cutoff plays a crucial role in distance-dependent statistical potential. Generally, a lower distance cutoff is better for native recognition, while a higher one is favorable for decoy discrimination. We developed an atomic angle- and distance-dependent potential (ANDIS) with distance cutoff being an adjustable parameter. ANDIS’s ability of native recognition is remarkably promoted by introducing the “effective atomic interactions”. Most of the native structures that fail to be recognized are small proteins or with poor MolProbity score. A distance-dependent atom-pair potential with random-walk reference state is combined to ANDIS when distance cutoff is ≥10 Å, which successfully enhances ANDIS’s ability of decoy discrimination. The results of benchmark tests indicate that ANDIS outperforms other state-of-the-art potentials in both native recognition and decoy discrimination.

Moreover, we investigated the effects of protein dataset on potential’s performance. Datasets culled by different parameter sets don’t make a real difference on ANDIS’s performance, but the size of dataset should reach a certain level. A dataset with about 3000 structures is adequate for ANDIS to achieve the optimal performance in native recognition. While the size reduces to hundreds of structures for optimizing the ability of decoy discrimination. Why is there such a difference? What is the best size of a representative dataset? How is the limitation of a potential in information extraction? These interesting questions remain to be further explored.

## Additional file

Additional file 1: **Figure S1.** Effects of distance cutoff on ANDIS’s performance for different decoy sets. **Figure S2.** Effects of protein dataset on ANDIS’s performance for different decoy sets. (DOCX 222 kb)

## Abbreviations

CASP: The Critical Assessment of protein Structure Prediction experiments
PCC: the Pearson’s correlation coefficient
