# Supplementary material for: ANDIS: an atomic angle- and distance-dependent statistical potential for protein structure quality assessment
Source: BMC Bioinformatics. 2019 Jun 3;20:299. doi: 10.1186/s12859-019-2898-y (PMC6547486; doi:10.1186/s12859-019-2898-y)
Supplement: Supplementary file 1 — Figure S1. Effects of distance cutoff on ANDIS’s performance for different decoy sets. Figure S2. Effects of protein dataset on ANDIS’s performance for different decoy sets. (DOCX 222 kb) [file 12859_2019_2898_MOESM1_ESM.docx]

ANDIS: an atomic angle- and distance-dependent statistical potential for protein structure quality assessment

Zhongwang Yu^1^, Yuangen Yao^1^, Haiyou Deng^1,2*^, and Ming Yi^1,2*^

1 Department of Physics, College of Science, Huazhong Agricultural University, Wuhan, 430070, China

2 Institute of Applied Physics, Huazhong Agricultural University, Wuhan 430070, China

**SUPPLEMENTAL INFORMATION**


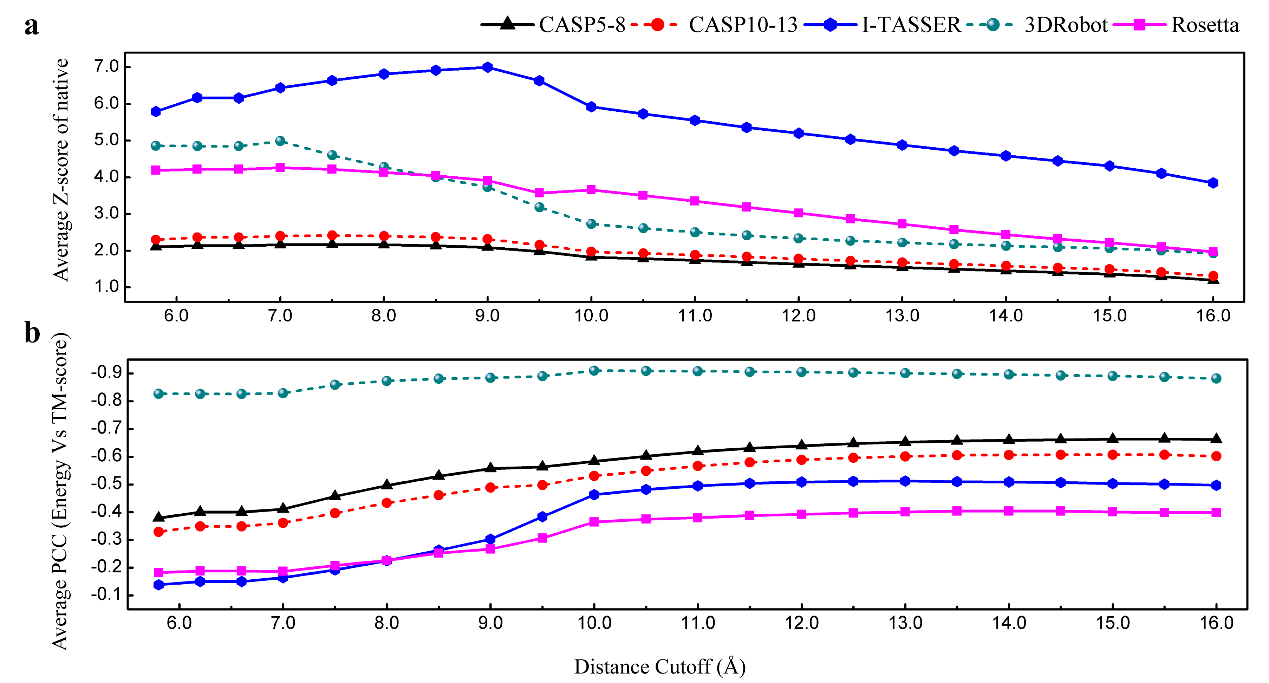


**Figure S1. Effects of distance cutoff on ANDIS’s performance for different decoy sets.**

**a.** effects of distance cutoff on ANDIS’s performance of native recognition; **b.** effects of distance cutoff on ANDIS’s performance of decoy discrimination.


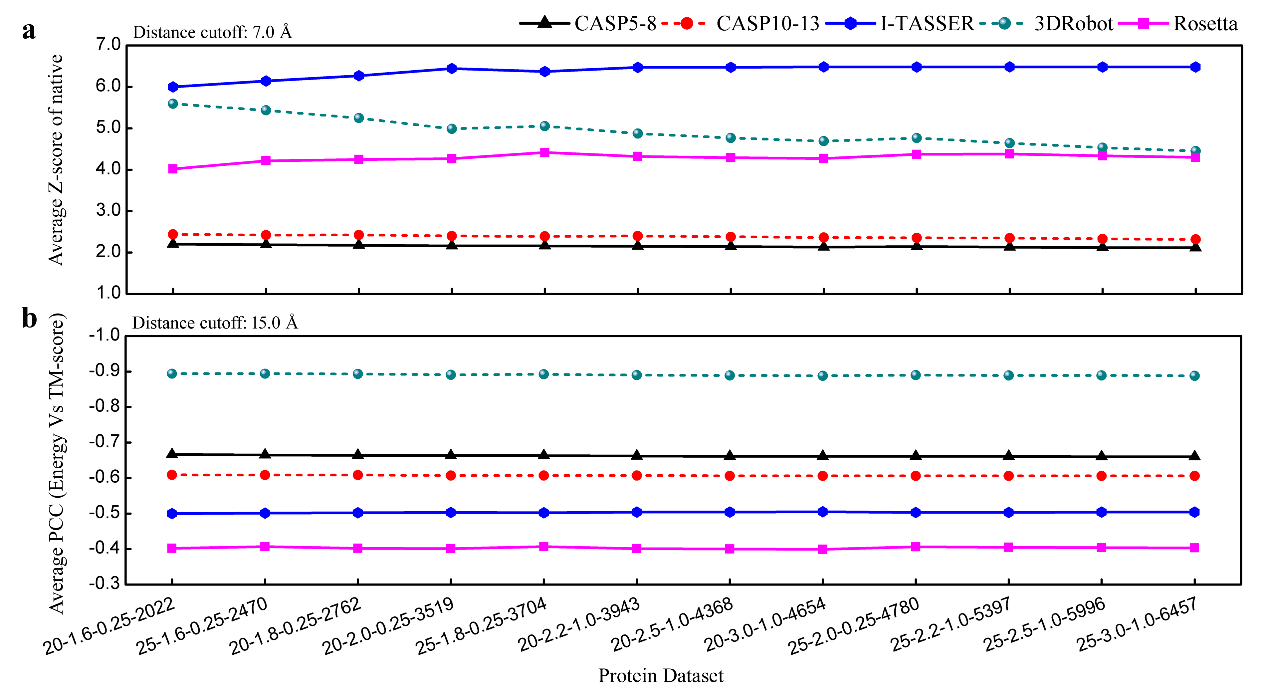


**Figure S2. Effects of protein dataset on ANDIS’s performance.**

**a.** effects of protein dataset on ANDIS’s performance of native recognition; **b.** effects of protein dataset on ANDIS’s performance of decoy discrimination. The horizontal axis corresponds to the potentials based on different protein datasets (e.g., 20-3.0-1.0-4654 refers to a dataset of 4654 protein structures with pairwise sequence identity <20%, resolution <3.0 Å and R-factor <1.0).
